# Exercise training with dietary restriction enhances circulating irisin level associated with increasing endothelial progenitor cell number in obese adults: an intervention study

**DOI:** 10.7717/peerj.3669

**Published:** 2017-08-14

**Authors:** Junhao Huang, Shen Wang, Fengpeng Xu, Dan Wang, Honggang Yin, Qinhao Lai, Jingwen Liao, Xiaohui Hou, Min Hu

**Affiliations:** 1 Guangdong Provincial Key Laboratory of Sports and Health Promotion, Scientific Research Center, Guangzhou Sport University, Guangzhou, China; 2 Department of Sports and Health, Guangzhou Sport University, Guangzhou, China; 3 School of Kinesiology, Shanghai University of Sport, Shanghai, China

**Keywords:** Exercise, Endothelial function, Obesity, Endothelial progenitor cells, Diet, Irisin

## Abstract

**Objective:**

Circulating endothelial progenitor cells (EPCs) correlate negatively with obesity. Previous studies have shown that exercise significantly restores circulating EPC levels in obese people; however, the underlying mechanisms have not been elucidated. Recently, irisin has been reported to have a critical role in the regulation of EPCs. This exercise-induced myokine has been demonstrated to play a therapeutic role in obesity. In this study, we hypothesized that the increase in circulating irisin may form a link with increasing EPC levels in obese people after exercise.

**Methods:**

Seventeen obese adults completed an 8-week program of combined exercise and dietary intervention. Clinical characteristics, blood biochemistry, and circulating irisin levels of subjects were measured before and after eight weeks of training. EPC levels were evaluated via flow cytometry, and EPC migratory and adhesive functions were also determined.

**Results:**

Circulating irisin levels significantly increased following the 8-week training program (*P* < 0.05). We furthermore observed an improvement in EPC numbers (*P* < 0.05), and EPC migratory and adhesive functions (*P* < 0.001 and *P* < 0.05, respectively) after the intervention. Additionally, we detected a positive correlation between changes in irisin and changes in EPC number (*r* = 0.52, *P* < 0.05).

**Discussion:**

For the first time, a positive correlation between increasing irisin levels and increasing EPC levels has been reported after an 8-week program, consisting of exercise and dietary intervention. This result suggests a novel effect of irisin on the regulation of EPC mobilization, which might contribute to improvement of endothelial function in obese people.

## Introduction

Vascular endothelial function is essential for the appropriate maintenance of cardiovascular health in humans (Ribeiro et al., 2010; Yang et al., 2007). An increasing body of evidence indicates that circulating bone marrow-derived endothelial progenitor cells (EPCs) play an important role in the regulation of endothelial function (Hill et al., 2003; Koutroumpi et al., 2012; Steiner et al., 2005; Tao et al., 2006). It has been demonstrated that circulating EPCs can increase neovascularization, repair endothelial injuries, and improve endothelial function (Koutroumpi et al., 2012; Steiner et al., 2005). A decrease in circulating EPCs indicates impaired endothelial function, and consequently, the number of serum EPCs can be used as a surrogate index of cumulative cardiovascular risk (Koutroumpi et al., 2012). In fact, Muller-Ehmsen et al. (2008) reported that obesity is associated with a reduction of the number of circulating progenitor cells. Further studies revealed evidence in support for a decrease in both number and function of EPCs in obese participants (Heida et al., 2010; MacEneaney et al., 2009; Tobler et al., 2010).

Both exercise and relevant dietary changes have independently been recognized as effective approaches to improve endothelial function (Brook, 2006; Sasaki et al., 2002; Sawyer et al., 2016; Woo et al., 2004). Moreover, research has been conducted on the effect of physical exercise or dietary restrictions on EPC mobilization, which resulted in interesting findings. For example, endurance exercise had a positive effect on EPC mobilization by enhancing both number of EPCs as well as their functions (Cesari et al., 2012; Schlager et al., 2011; Van Craenenbroeck et al., 2010). In addition, a 10-month treatment regimen consisting of supervised diet and physical training was effectively increasing EPC levels in obese adolescents (Bruyndonckx et al., 2015). Importantly, a combination of exercise and diet may enlarge the effect on EPC levels compared to each single intervention alone. For example, in subjects with the metabolic syndrome, a 12-week moderate-to-high-intensity endurance training combined with a hypocaloric Mediterranean diet produced a more pronounced increase in EPC levels than a diet-only program (Fernandez et al., 2012). This indicates that it would be an advantageous strategy to improve EPC mobilization by combining exercise and dietary intervention. However, the mechanisms underlying effects of lifestyle interventions on EPCs that include exercise and diet have not been elucidated to date.

Irisin, a recently discovered exercise-induced myokine, is a cleavage product of fibronectin type III domain containing 5 (FNDC5) produced in response to the activation of peroxisome proliferator-activated receptor- γ coactivator-1 alpha (PGC-1α). Previous studies have shown that irisin converts white adipocytes into brown adipocytes (Bostrom et al., 2012). Thus, irisin is considered to play a therapeutic role in type 2 diabetes and obesity due to the thermogenic changes in white adipose tissue (Bostrom et al., 2012; Kelly, 2012; Villarroya, 2012).

Although the effects of exercise on circulating irisin in humans are still controversial, several studies reported that exercise promotes irisin secretion (Bluher et al., 2014; Bostrom et al., 2012; Huh et al., 2014; Kim et al., 2016; Norheim et al., 2014). However, it seems that dietary modification has a small effect on serum irisin levels in healthy humans (Park et al., 2014). In addition, increasing evidence indicates that irisin might be involved in the regulation of endothelium-dependent vasorelaxation in diabetes and obesity (Hou, Han & Sun, 2015; Xiang et al., 2014). Additionally, a recent study reported that irisin restored both the number and function of EPCs via the PI3K/Akt/eNOS pathway in diabetic mice (Zhu et al., 2016). However, currently, no reports are available on the relationship between irisin and EPCs under the lifestyle modifications of exercise and dietary intervention.

Thus, in the present study, we investigated the effect of an 8-week intervention program of a combination of exercise and dietary restriction on circulating irisin concentrations, and their relationship with the change in EPC levels in an obese population. We hypothesized that the increase in circulating irisin was correlated with a corresponding increase in EPC levels in obese subjects after eight weeks of exercise and dietary intervention.

## Materials and Methods

### Participants

Participants aged between 18 and 40 years were recruited from the Shenzhen Sunstarasia Weight Loss Camp. Participants were included that met the following initial eligibility requirements: (a) obesity status, as assessed by body mass index (BMI) ≥30 kg/m^2^; (b) absence of unstable angina pectoris, cardiomyopathy, severe lung diseases, or renal failure. Informed consent was obtained from each individual prior to measurements. This study was conducted according to the Declaration of Helsinki and was approved by the Ethics Committee of Guangzhou Sport University (approval No. GSU20160012). Participant recruitment and follow-up were conducted between March 2016 and September 2016. The trial was registered in ISRCTN registry (ISRCTN83594346). The protocol for this trial and supporting TREND checklist are available as supporting information (see S1 Protocol and S1 Checklist).

Eligible participants from a traditional weight loss camp (located at a remote district of Huizhou city, Guangdong, China) self-selected to join this program. The closed camp provided uniformly controlled accommodation, diet, and physical training during an 8-week intervention period. The camp participants were housed in the same building and could not come and go freely during their stay. The location, residential setting, and level of obesity (BMI = 37.8 ± 5.0 kg/m^2^) prevented identification of an appropriate control group.

### Diet restriction

Participants were provided with calorie-restricted diets that contained 1,300–2,200 kcal/day based on their weight. During the study, the menu was changed weekly, and the diet was adjusted each week according to an individual’s updated weight. The energy percentages provided by protein, fat, and carbohydrate were 20%, 20%, and 60%, respectively. Energy distributions at breakfast, lunch, and dinner were 30%, 40%, and 30%, respectively. All meals were prepared and supervised by registered professional dietitians during the diet intervention.

### Exercise training

Subjects performed a training program six days/week for eight weeks. The program was primarily comprised of endurance exercise such as bicycling, walking, running, dancing, and ball games for five hours/day. It was also supplemented by strength training. The endurance exercises involved an equivalent combination of moderate (i.e., four metabolic equivalents of tasks (METs)) and high intensity (eight METs) physical training. We used the following equation to calculate the exergy expenditure during activity (Pinheiro Volp et al., 2011): energy expenditure (kcal/min) = 0.0175 × METs × weight (kg). The exercise program was specifically designed to induce an energy expenditure of 1,500–2,500 kcal/day. The intensity of moderate-intensity exercise was set at 70–85% of the subject’s maximum heart rate (HR_max_), which was calculated via the formula of 208 − (0.7× age). Heart rate was continuously monitored by Polar heart rate monitors and recorded by researchers. The high-intensity training (∼90% of HR_max_) was alternated with low-intensity exercise (∼60% of HR_max_) during training. Strength training was implemented at 40–50% maximal strength for 2–3 sets of 12–15 RM with 2–3 min of rest between sets. Qualified trainers supervised the subjects during the program. All measurements were obtained before and after the 8-week training program.

The primary outcome measure for this study was endothelial function, which was assessed via flow-mediated dilation (FMD). Secondary outcomes were anthropometry, resting heart rate, blood pressure, body composition, aerobic fitness, maximal strength, EPC number and function, as well as multiple biochemical markers (lipid profiles, insulin, tumor necrosis factor-alpha, high-sensitivity C-reactive protein, superoxide dismutase, vascular endothelial growth factor, endothelial nitric oxide synthase, adiponectin, and irisin).

### Anthropometry and body composition

Height and weight were measured to calculate the BMI (kg/m^2^). Body composition was determined using a body composition analyzer (Inbody 370; Biospace, Seoul, Korea).

### Resting heart rate and blood pressure

After subjects rested for at least 10 min, resting heart rate and peripheral brachial systolic/diastolic blood pressure were measured in triplicate. The average of three readings was recorded.

### Aerobic fitness

Aerobic fitness was assessed using the Physical Working Capacity test on a cycle ergometer (Ergoselect 100; Ergoline, Bitz, Germany) at a heart rate of 150 or 170 beats/min (PWC150 or PWC170). Seat height was adjusted to individual satisfaction of each subject, and toe clips with straps were used to prevent feet from slipping off the pedals. Prior to the tests, subjects were instructed that they should pedal with a steady cadence of 60 revolutions/min. The start power was 50 watts (W) and was followed by a gradual increase of 25 W for PWC150 or 50 W for PWC170 every 2 min until the targeted heart rate (150 or 170 beats/min, respectively) was achieved and maintained at a steady state. PWC150 or PWC170 were calculated as the power corresponding to heart rate of 150 or 170 beats/min and expressed as W per kg of body mass (W/kg).

### Maximum strength test

The maximum strength was determined by a one-repetition maximum (1RM) bench press test, which was administered after each subject performed two warm-up sets. After all warm-up sets were completed, the subject then attempted the 1RM.

### Endothelial function

Subjects were requested to fast, abstain from exercise and the consumption of alcohol and caffeine, and withhold all medications and supplements known to affect vascular function for at least 12 h prior to testing. They were asked to rest in a quiet and air-conditioned room (22–25 °C) in a supine position for 30 min right before examination. Ultrasound equipment and a high-resolution linear array transducer coupled to computer-assisted analysis software provided one longitudinal and two short-axis images using a 10 MHz H-type probe (UNEXEF38G; UNEX, Nagoya, Japan). This was used to scan the brachial artery in B-mode 5 to 10 cm above the right elbow. This location was marked on the skin of each participant and all subsequent measurements were performed at the same location. When the clearest B-mode image of the intima-media complex had been obtained, a stereotactic probe holder held the transducer at the same point throughout the scan. FMD was measured via A-mode waves as a signal of the intima-media complex that was synchronized with the electrocardiographic R-waves and automatically tracked. After measuring baseline brachial artery diameter, we compressed the brachial artery (at least 50 mm Hg above systolic blood pressure) for 5 min, using a blood pressure cuff placed around the forearm. After compression, the maximum brachial artery diameter was measured after cuff release for 2 min (Miyagi et al., 2014; Stoner & Sabatier, 2012; Tomiyama et al., 2008). The brachial artery peak hyperemic shear rate was calculated as eight times the peak velocity divided by the diameter at the time of peak velocity.

FMD was calculated as the maximum percentage increase in arterial diameter during continuous measurement of arterial diameter following cuff deflation. Subsequently, endothelium-independent dilation was measured by sublingually administering 500 µg of nitroglycerine. The menstrual cycle of female subjects was recorded and they were asked to test on day 1–7 of the menstrual cycle. The same operator collected all measurements.

In our laboratory, we performed repeated FMD measurements on seven healthy adults (20–35 years old, 57% male). The intraclass correlation coefficient for repeated readings was *r* = 0.88 with a coefficient of variation of 11.4%.

### Blood markers

A fasting blood sample was collected into evacuated plastic tubes containing ethylenediaminetetraacetic acid (EDTA). Whole venous blood was also collected in tubes without anticoagulant for serum preparation. Total cholesterol, triglycerides, high-density lipoprotein cholesterol (HDL-c), low-density lipoprotein cholesterol (LDL-c), fasting glucose, and fasting insulin were measured. Insulin resistance was evaluated using the Homeostasis Model of Assessment of Insulin Resistance (HOMA-IR) and was calculated as (fasting insulin (µU/ml) × fasting glucose (mmol/L)) / 22.5. Serum concentrations of vascular endothelial growth factor (VEGF), endothelial nitric oxide synthase (eNOS), adiponectin, tumor necrosis factor-alpha (TNF-α), high-sensitivity C-reactive protein (hsCRP), and superoxide dismutase (SOD) were analyzed using ELISA Kits (Cusabio, Biotech. Co., Ltd., Wuhan, China), following the manufacturer’s instructions for each kit. Serum irisin concentrations were measured using an irisin ELISA kit (Cat. EK-067-29; Phoenix Pharmaceuticals, Burlingame, CA, USA), which had previously been validated via MS spectrometry analysis (Huh et al., 2015; Polyzos & Mantzoros, 2015; Zhang et al., 2014). Cross reactivity was validated via western blot. The minimum detectable concentration was 1.43 ng/ml. Intra- and interassay variations were below 10% and 15%, respectively (Benedini et al., 2017).

### Flow cytometric quantification of EPCs

EPCs are defined as CD34^+^/KDR^+^ cells. EDTA-anticoagulated whole blood samples (100 µl) were stained for 10 min at room temperature with APC-labeled anti-human CD34 (eBioscience, San Diego, CA, USA) and AlexaFlour488-labeled anti-human KDR (BioLegend, San Diego, CA, USA) monoclonal antibodies. Fluorescent isotype-matched antibodies were used as controls. EPCs were measured using a Cytomics FC500 flow cytometer (Beckman Coulter, Brea, CA, USA), and acquisition was stopped after 40,000 events. Data were analyzed using CXP software 2.0 (Fernandes et al., 2012).

### EPC culture

EPCs were isolated and cultured, following previously described protocols (Fernandes et al., 2012; Junhui et al., 2008; Tobler et al., 2010). Briefly, peripheral blood (10 ml) was obtained from obese subjects, and total mononuclear cells were isolated via density gradient centrifugation (400 g for 30 min) with Histopaque-1077 (Sigma, St. Louis, MO, USA). Then, cells were cultured on fibronectin-coated 6-well plates in M199 medium supplemented with 20% FBS, 100 U/ml penicillin, 100 µg/ml streptomycin, and 10 ng/ml VEGF. After four days in culture, nonadherent cells were removed. Adherent cells were maintained until day seven and then used for EPC functional assays.

**Figure 1:**
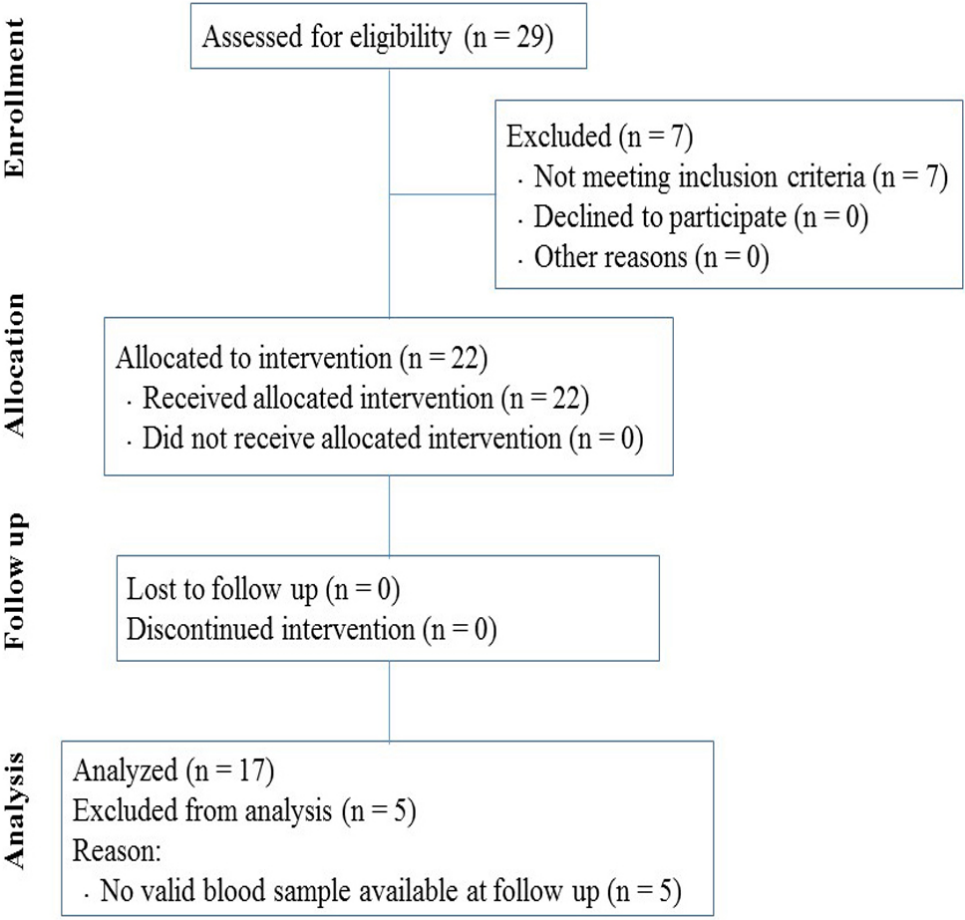
Flow diagram of participants through the study.

### EPC migration assay

EPC migration was determined with a modified Boyden chamber (Costar Transwell assay, 8 µm pore size; Corning, NY, USA) (Junhui et al., 2008; Xia et al., 2012). Briefly, a total of 2 × 10^4^ isolated EPCs were resuspended in 250 µl serum-free M199 medium and placed in the upper chamber. The chamber was placed in a 24-well culture dish containing 500 µl M199 medium supplemented with 10 ng/ml VEGF. After a 24-hour incubation at 37 °C, the membrane was briefly washed with phosphate-buffered saline (PBS) and fixed with 4% paraformaldehyde. The membrane was then stained using 0.1% crystal violet solution and carefully removed. The transmigrated cells were manually counted in three random microscopic fields (×200) by independent, blinded investigators.

### EPC adhesion assay

The EPC adhesion assay was conducted according to previously described techniques (Junhui et al., 2008; Xia et al., 2012). Briefly, EPCs were isolated and resuspended in M199 medium with 5% FBS. An equivalent amount of cells were placed on a fibronectin-coated 96-well plate and incubated at 37 °C in 5% CO_2_ for 6 h. After two gentle washes with PBS, adherent cells were counted.

### Statistical analysis

Analyses were performed using SPSS 16.0 (SPSS Inc., Chicago, IL, USA). Paired-sample *t*-tests were used to compare the effects of an 8-week intervention of exercise and diet on different variables. Pearson’s correlation was calculated to determine associations. Values are reported as mean ± SD. Sample size was calculated based on a previous study that reported a 1.11% change in FMD after each 10 kg weight loss (Joris, Zeegers & Mensink, 2015), with an expected within-subject SD of 1.3% in FMD from our laboratory. The sample size required for the study (with an α of 0.05 and β of 0.20 at 5% level of significance (two-sided) and estimating a refusal rate of 20%) was 20 subjects. Cohen’s d was used to calculated effect sizes on pairwise comparisons, with 0.2, 0.5, and 0.8, representing small, medium, and large effects, respectively (Mullineaux, Bartlett & Bennett, 2001). A *P*-value <0.05 was used to indicate statistical significance.

## Results

### Participants

The flow of participants through each stage of the intervention is shown in Fig. 1 according to the TREND statements. Of the 29 initially enrolled subjects, 22 met the criteria for inclusion determined in the study and were selected for the research program. We only analyzed data from subjects that completed the program and provided valid measurements at both baseline and follow-up.

### Anthropometry and body composition

To determine how the exercise and diet intervention affected overall health, we measured body composition. As shown in Table 1, body weight significantly decreased from 116.6 ± 24.6 kg to 103.4 ± 22.4 kg (*P* < 0.001, *d* =  − 0.56). BMI decreased from 37.8 ± 5.0 kg/m^2^ to 33.5 ± 4.4 kg/m^2^ (*P* < 0.001, *d* =  − 0.91) following the 8-weeks of exercise and dietary intervention. We also observed a decrease in body fat mass from 47.4 ± 12.7 kg to 38.2 ± 12.2 kg (*P* < 0.001, *d* =  − 0.74) following the intervention (Table 1). Furthermore, body fat percentage decreased from 40.9 ± 4.6% to 36.6 ±5.9% (*P* < 0.001, *d* =  − 0.81) after the intervention (Table 1). These results suggest that the intervention was effective in reducing body fat and improving BMI in obese subjects.

**Table 1:** Subject characteristics and parameters of anthropometry, body composition, resting heart rate, blood pressure, peak brachial artery shear rate, aerobic fitness and maximal strength before and after 8-week combined exercise and diet intervention.

Parameters	Before	After
Age (yr)	22.1 ± 2.8	–
Height (cm)	174.8 ± 7.64	–
Gender (n) (M/F)	17(11/6)	–
Body weight (kg)	116.6 ± 24.6	103.4 ± 22.4^***^
BMI (kg/m^2^)	37.8 ± 5.0	33.5 ± 4.4^***^
Body fat (kg)	47.4 ± 12.7	38.2 ± 12.2^***^
Body fat (%)	40.9 ± 4.6	36.6 ± 5.9^***^
Resting heart rate (bpm)	74 ± 9	61 ± 9^***^
SBP (mmHg)	127.8 ± 11.8	115.3 ± 7.4^***^
DBP (mmHg)	85.2 ± 9.4	79.9 ± 10.8
Peak shear rate (s^−1^)	141.0 ± 84.1	177.3 ± 128.3
PWC150 (W/kg, *n* = 9)	1.38 ± 0.41	1.74 ± 0.32^***^
PWC170 (W/kg, *n* = 8)	1.99 ± 0.50	2.65 ± 1.03
1RM bench press (lbs)	133 ± 61	140 ± 60

**Notes.**

BMI body mass index SBP systolic blood pressure DBP diastolic blood pressure PWC150 Physical Working Capacity at a heart rate of 150 bpm PWC170 Physical Working Capacity at a heart rate of 170 bpm 1RM one repetition maximum

Values are presented as mean ± SD.

*** *P* < 0.001 vs. Before.

### Resting heart rate and blood pressure

The intervention of exercise and diet significantly decreased the resting heart rate from 74 ± 9 bpm to 61 ± 9 bpm (*P* < 0.001, *d* =  − 1.44) and significantly decreased systolic blood pressure from 127.8 ± 11.8 mmHg to 115.3 ± 7.4 mmHg (*P* < 0.001, *d* =  − 1.27) (Table 1). However, no significant change was detected in diastolic blood pressure (before: 85.2 ± 9.4 mmHg vs. after: 79.9 ± 10.8 mmHg, *P* > 0.05) (Table 1).

### Aerobic fitness and maximal strength

We observed an increase in PWC150 from 1.38 ± 0.41 W/kg to 1.74 ± 0.32 W/kg (*P* < 0.001, *d* = 0.98) following the intervention. Interestingly, although PWC170 had an increasing trend from 1.99 ± 0.50 W/kg to 2.65 ± 1.03 W/kg after intervention, but this trend did not reach significance (*P* = 0.06) (Table 1). There was also no significant change in 1RM bench press resulting from the intervention (before: 133 ± 61 lbs vs. after: 140 ± 60 lbs, *P* > 0.05) (Table 1).

**Table 2:** Parameters of blood markers before and after 8-week combined exercise and diet intervention.

Parameters	Before	After
Cholesterol (mmol/l)	5.35 ± 0.92	4.74 ± 1.34^*^
Triglycerides (mmol/l)	2.15 ± 1.08	1.32 ± 0.88^**^
HDL-c (mmol/l)	0.99 ± 0.14	0.99 ± 0.22
LDL-c (mmol/l)	3.44 ± 0.75	2.99 ± 0.95^*^
Fasting blood glucose (mg/dl)	5.61 ± 0.56	5.39 ± 0.57
Fasting insulin (pmol/l)	200.8 ± 115.8	116.7 ± 81.2^**^
HOMA-IR	7.34 ± 4.9	4.08 ± 2.9^**^
Irisin (ng/ml)	43.9 ± 11.0	53.9 ± 13.4^*^
VEGF (pg/ml)	27.6 ± 16.11	43.0 ± 27.36^*^
eNOS (IU/ml)	150.0 ± 138.73	530.8 ± 259.88^***^
Adiponectin (ng/ml)	16.8 ± 14.98	32.5 ± 23.25^*^
TNF-α (pg/ml)	453.3 ± 274.65	201.6 ± 70.04^**^
hsCRP (µg/ml)	2.49 ± 1.71	1.36 ± 1.61^**^
SOD (IU/ml)	0.63 ± 0.34	1.40 ± 0.66^***^

**Notes.**

HDL-c high-density lipoprotein cholesterol LDL-c low-density lipoprotein cholesterol HOMA-IR Homeostasis Model of Assessment of Insulin Resistance VEGF vascular endothelial growth factor eNOS endothelial nitric oxide synthase TNF-α tumor necrosis factor-alpha hsCRP high-sensitivity C-reactive protein SOD superoxide dismutase

Values are presented as mean ± SD.

* *P* < 0.05.

** *P* < 0.01.

*** *P* < 0.001 vs. Before.

### Blood chemistry

Blood lipid levels can indicate a risk of pathological diseases, such as atherosclerosis and diabetes. Thus, we measured the changes in circulating lipids following the intervention. We observed a significant decrease in the serum levels of cholesterol (before: 5.35 ± 0.92 mmol/l vs. after: 4.74 ± 1.34 mmol/l, *P* < 0.05, *d* =  − 0.53), triglycerides (before: 2.15 ± 1.08 mmol/l vs. after: 1.32 ± 0.88 mmol/l, *P* < 0.01, *d* =  − 0.84), LDL-c (before: 3.44 ± 0.75 mmol/l vs. after: 2.99 ± 0.95 mmol/l, *P* < 0.05, *d* =  − 0.53), and fasting insulin (before: 200.8 ± 115.8 pmol/l vs. after: 116.7 ± 81.2 pmol/l, *P* < 0.01, *d* =  − 0.84) (Table 2). Analysis of insulin resistance was measured via HOMA-IR. Our results revealed that insulin resistance significantly decreased after the intervention (*P* < 0.01, *d* =  − 0.81) (Table 2). Although we observed a decreasing trend in fasting blood glucose from 5.61 ± 0.56 mg/dl to 5.39 ± 0.57 mg/dl after the intervention, this change did not reach significance (*P* > 0.05). There was no significant change in HDL-c levels resulting from the intervention (before: 0.99 ± 0.14 mmol/l vs. after: 0.99 ± 0.22 mmol/l, *P* > 0.05) (Table 2). We also measured circulating adiponectin because it is an adipocyte-secreted hormone associated with improved insulin sensitivity and the amelioration of the metabolic syndrome. We found that diet and exercise significantly increased adiponectin levels in obese subjects (*P* < 0.05, *d* = 0.80). Two important markers for endothelial function are VEGF and eNOS, and both VEGF and eNOS were significantly improved after the intervention. As shown in Table 2, we also observed a significant increase in circulating levels of irisin after the intervention (before: 43.9 ± 11.0 ng/ml vs. after: 53.9 ± 13.4 ng/ml, *P* < 0.05, *d* = 0.82). Moreover, the intervention of diet and exercise markedly improved inflammation and oxidative stress, which was reflected in a reduction of TNF-α and hsCRP levels and an increase in SOD levels (Table 2).

### Endothelial function

**Figure 2:**
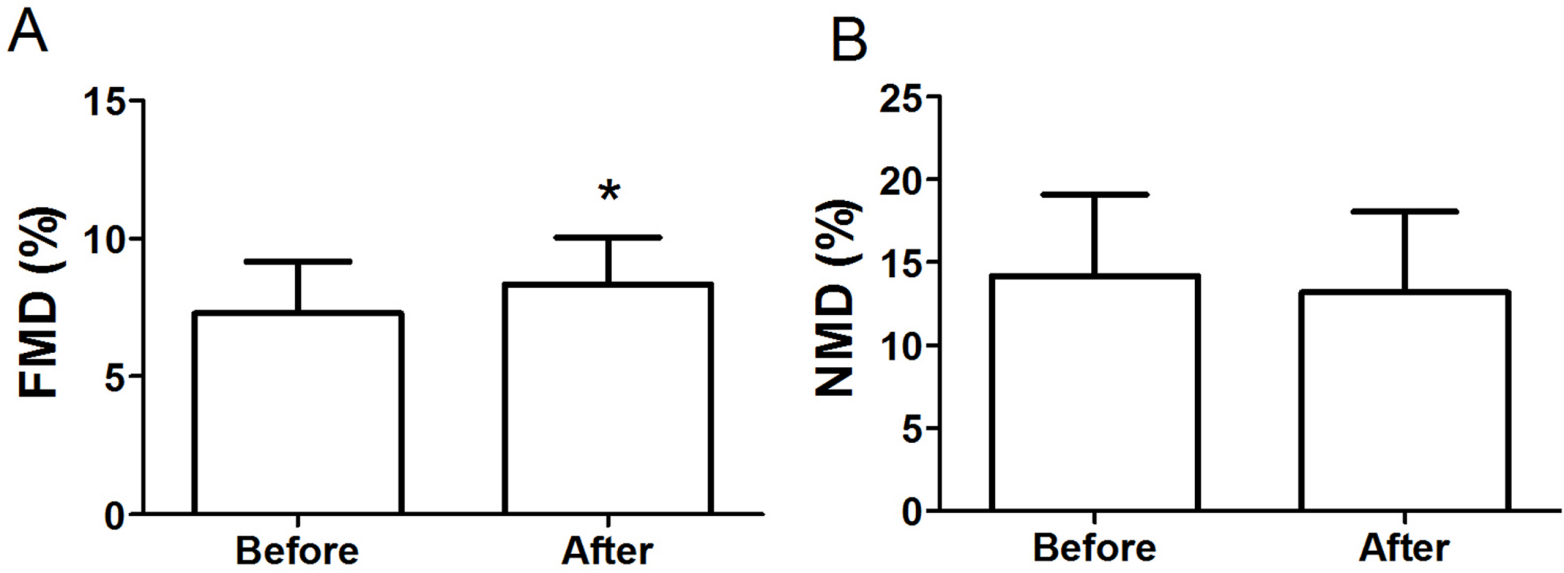
FMD (A) and NMD (B) in obese subjects before and after 8-week combined exercise and diet intervention. FMD, flow-mediated dilation; NMD, nitroglycerine-mediated dilation. Values are presented as mean ± SD. ^*^P < 0.05 vs. Before.

Figure 2A shows that FMD significantly improved from 7.28 ± 1.89% to 8.33 ± 1.71% (*P* < 0.05, *d* = 0.58) after the intervention. The brachial artery peak hyperemic shear rate did not differ in response to the intervention (before: 141.0 ± 84.1 s^−1^ vs. after: 177.3 ± 128.3 s^−1^, *P* > 0.05) (Table 1). Moreover, the intervention had no significant impact on endothelium-independent dilation induced by nitroglycerine (before: 14.16 ± 4.96% vs. after: 13.18 ± 4.87%, *P* > 0.05) (Fig. 2B).

### Circulating EPC levels

**Figure 3:**
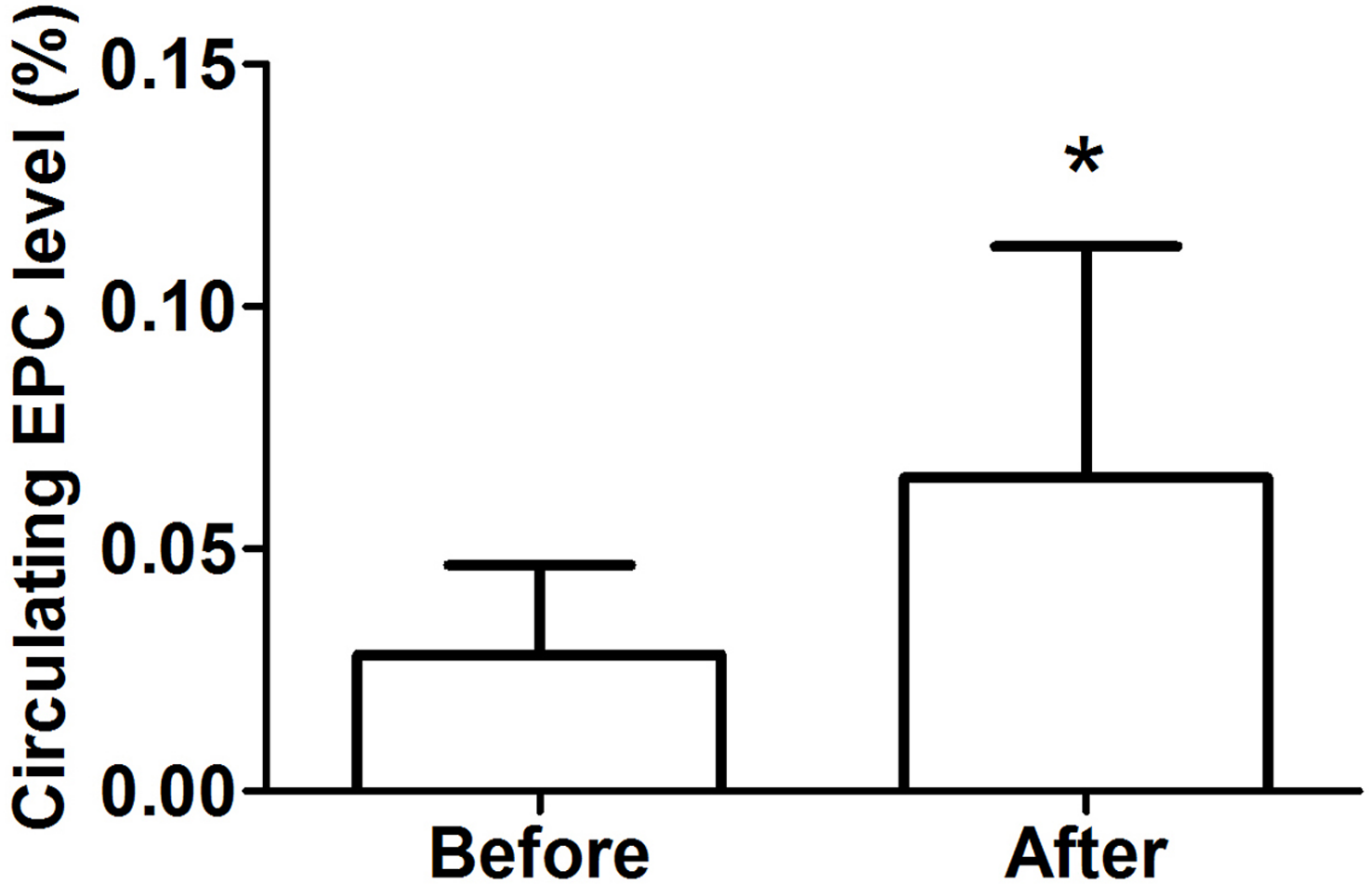
Circulating EPC levels in obese subjects before and after 8-week combined exercise and diet intervention. Values are presented as mean ± SD. ^*^P < 0.05 vs. Before.

As shown in Fig. 3, we detected a significant increase in circulating EPCs after the intervention (before: 0.028 ± 0.018% vs. after: 0.065 ± 0.048%, *P* < 0.05, *d* = 1.02).

### EPC functions

**Figure 4:**
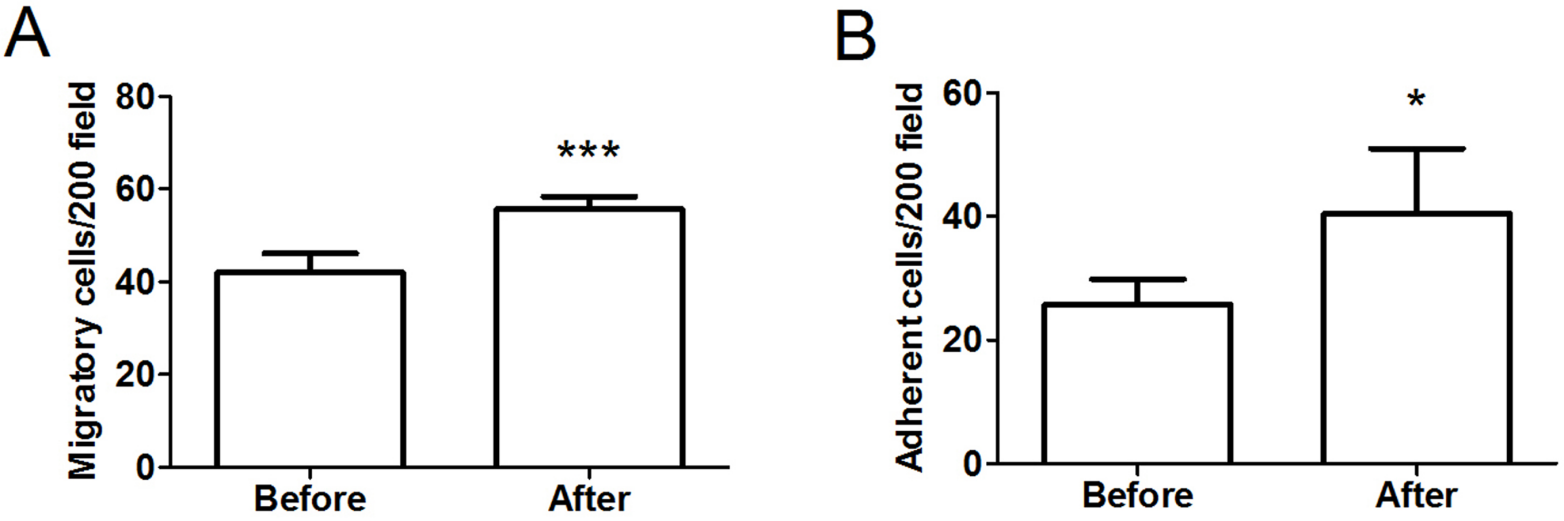
Migratory (A) and adhesive (B) capacities of EPCs in obese subjects before and after 8-week combined exercise and diet intervention. Values are presented as mean ± SD. ^*^P < 0.05, ^***^P < 0.001 vs. Before.

To assess the functional activity of EPCs, we determined the migration of isolated EPCs in response to VEGF using a modified Boyden chamber. As illustrated in Fig. 4A, there was a significant improvement in the migratory capacity of EPCs after intervention (before: 42.0 ± 4.08 cells/ ×200 field vs. after: 55.67 ± 2.73 cells/ ×200 field, *P* < 0.001, *d* = 3.93). Moreover, we assessed the adhesive capacity of EPCs because adhesion to the extracellular matrix is believed to be important during new blood vessel growth. As shown in Fig. 4B, the intervention also significantly improved EPC adhesive function (before: 25.75 ± 4.11 cells/ ×200 field vs. after: 40.50 ± 10.46 cells/ ×200 field, *P* < 0.05, *d* = 1.86).

### Correlations

**Figure 5:**
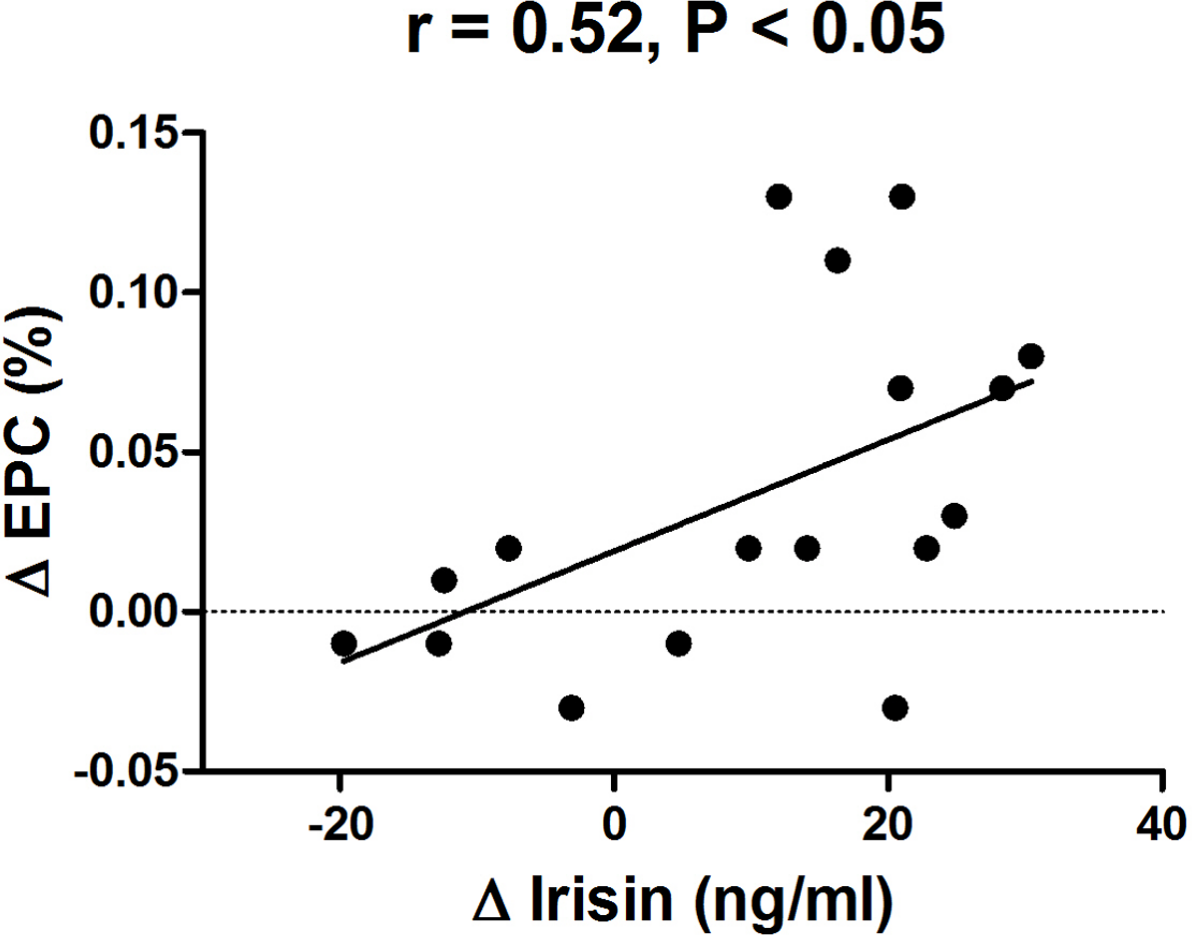
Correlation between change in circulating irisin levels and change in circulating EPC number in obese subjects after 8-week combined exercise and diet intervention. Values are presented as mean ± SD. *r* = 0.52, *P* < 0.05.

A significant correlation between the increase of circulating irisin levels and the increase in the number of EPCs was observed by the intervention (*r* = 0.52, *P* < 0.05) (Fig. 5).

## Discussion

This study showed that circulating irisin levels had significantly increased in obese subjects after an 8-week program consisting of exercise and dietary intervention. Furthermore, the increase in irisin was positively correlated with an increase of EPC levels. These findings suggest that irisin may be involved in the regulation of EPC mobilization due to lifestyle modifications that include exercise and diet. To our knowledge, this is the first report to investigate the relationship between circulating irisin and EPC levels in an obese population.

Recently, a role for EPCs in endothelial repair was discovered, and EPCs have been identified as an independent determinant of endothelial function. A significant elevation in EPC was previously shown in obese adolescents after 12 weeks of exercise (Park et al., 2012). Moreover, it has also been reported that dietary intervention can increase EPC numbers in obese subjects (Heida et al., 2010). In the current study, our results revealed a significant increase in the number of circulating EPCs in obese subjects after exercise and dietary intervention. Moreover, we also examined the effect of eight weeks of weight loss therapy on the functional capacity of EPCs. Our results demonstrated that the migration and adhesion of EPCs improved following the combined exercise and diet intervention. These results are in accordance with a recent study that reported that circulating EPC levels in obese adolescents were increased after a treatment program consisting of moderate diet and exercise training (Bruyndonckx et al., 2015).

Irisin is a signaling protein secreted by skeletal muscles, which is released into the circulation after proteolysis of the membrane protein FNDC5 (Hou, Han & Sun, 2015). Although the effects of exercise on circulating irisin in humans remain controversial, several studies have demonstrated that exercise promotes irisin secretion (Bostrom et al., 2012; Huh et al., 2014; Kim et al., 2016; Norheim et al., 2014). In this study, we furthermore found that serum irisin levels were significantly increased after exercise and dietary intervention.

Recent studies have found an important role of irisin in the regulation of endothelial function in people suffering from obesity or diabetes (Hou, Han & Sun, 2015; Xiang et al., 2014). However, the underlying mechanisms remain unknown. In this study, we hypothesized that the increase of circulating irisin levels might correlate with the increase in EPC levels after exercise and dietary intervention. Our data confirmed an association between changes in circulating irisin and changes in EPC levels in obese subjects after weight loss intervention. This result was consistent with a recent study, which demonstrated that exogenous administration of irisin was able to restore the number and functional capacityof EPCs via the PI3K/Akt/eNOS pathway in diabetic mice (Zhu et al., 2016). Irisin is an exercise-induced myokine; therefore, it could be activated in response to exercise training. This would thereby exert its role in promoting EPC mobilization, which may in turn contribute to improved endothelial function in obese people. Future studies are warranted to elucidate the complex association between exercise training, exercise-induced irisin levels, and changes in EPC number and function in humans and animals with obesity.

We recognize the limitations of the present study. For example, we did not have a control group because the camp program was a traditional residential camp with a specific aim of weight loss. The camp was located in a remote district of the city, and subjects enrolled in the program came from all over the country and had an average BMI over 35 kg/m^2^ (37.8 ± 5.0 kg/m^2^). Therefore, factors such as location, the level of obesity, and residential aspects restricted the opportunity to select an appropriate control group. This has been previously described elsewhere (Gately et al., 2000). The lack of a designated control group allows a less clear interpretation of the results. The results observed in this study will require confirmation in randomized controlled trials. Furthermore, another limitation of this study was the small sample size. Due to the strength and endurance needed for the strict, military-style camp for eight weeks, we were only able to enroll a limited number of qualified volunteers. Future studies on a larger scale will be performed. It will be also interesting to quantify the motor cortical changes following training with non-invasive measurement approaches, for instance (Huang et al., in press; Shen et al., 2017; Zhou et al., 2017).

In conclusion, this study found a positive correlation between an increase of circulating irisin and an increase in EPC levels after an 8-week program consisting of exercise and dietary intervention. This result suggests a beneficial effect of irisin on the regulation of EPC mobilization, which might contribute to an improvement in endothelial function in obese people. The underlying mechanism of association between irisin and EPCs needs to be investigated for future clinical and animal studies.

##  Supplemental Information

10.7717/peerj.3669/supp-1 Supplemental Information 1 S1 Study Protocol

10.7717/peerj.3669/supp-2 Supplemental Information 2 S2 Protocol in Chinese

10.7717/peerj.3669/supp-3 Supplemental Information 3 S1 Checklist

10.7717/peerj.3669/supp-4 Data S1 S1 Raw data

## Additional Information and Declarations

### Clinical Trial Registration

The following information was supplied regarding Clinical Trial registration: ISRCTN83594346.
