# Supplementary material for: Exercise training with dietary restriction enhances circulating irisin level associated with increasing endothelial progenitor cell number in obese adults: an intervention study
Source: PeerJ. 2017 Aug 14;5:e3669. doi: 10.7717/peerj.3669 (PMC5560232; doi:10.7717/peerj.3669)
Supplement: Supplemental Information 1 [file peerj-05-3669-s001.docx]

**
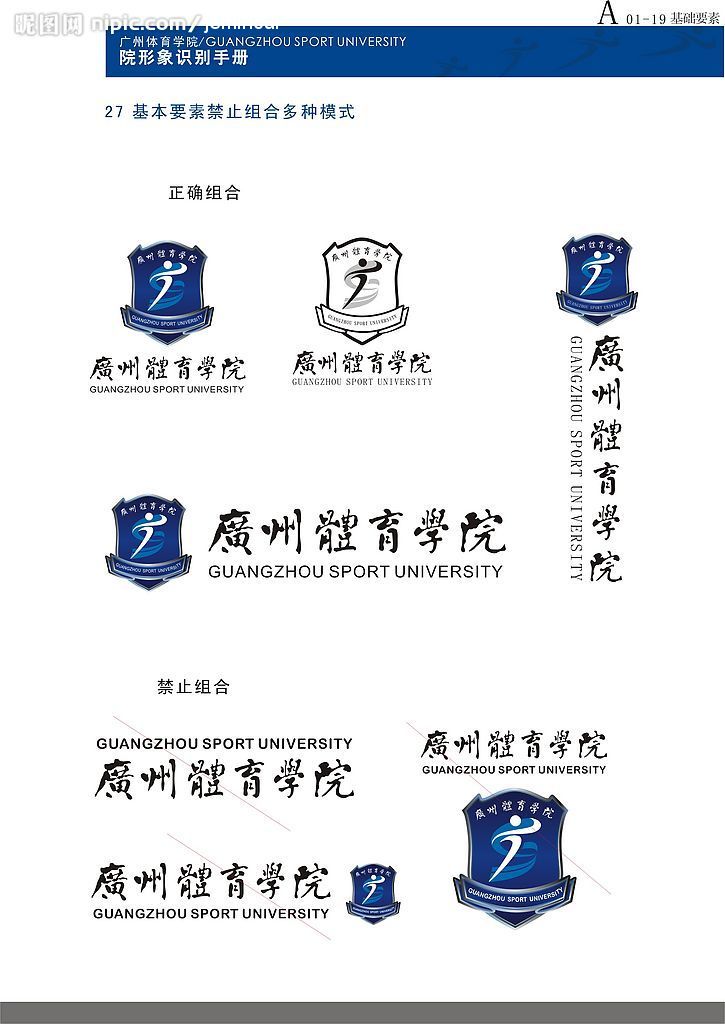
**

**地址：中国广州市广州大道中1268号 邮编：510500**

**Address: No. 1268, Middle Guangzhou Avenue, Guangzhou, China Zip code: 510050**

**Website: http://www.gipe.edu.cn/**

Title: Effects of exercise training and diet restriction on cardiovascular function in obese population: an intervention study

Short title: Exercise, diet and cardiovascular function

Protocol Number: Trial2016.1 Version 1.0

Sponsor: Guangzhou Sport University

1268 Middle Guangzhou Avenue

Guangzhou

China

Sponsor’s representative: Miss Dan Wang

1268 Middle Guangzhou Avenue

Scientific Research Building Floor 1

Guangzhou Sport University

Guangzhou

China

+86 (0)20 3802 5087

[17880383@qq.com](mailto:17880383@qq.com)

Sponsor’s Medical Expert: Dr. Junhao Huang

1268 Middle Guangzhou Avenue

Scientific Research Building Floor 1

Guangzhou Sport University

Guangzhou

China

+86 (0)20 3802 5087

[junhaohuang2006@hotmail.com](mailto:junhaohuang2006@hotmail.com)

Chief Investigator: Dr. Min Hu

1268 Middle Guangzhou Avenue

Guangzhou

China

+86 (0)20 3802 4222

[whoomin@hotmail.com](mailto:whoomin@hotmail.com)

Trail Site: Sunstarasia Weight Loss Camp

Huizhou

China

1. **Background**
   1. **Prevalence of obesity**

Obesity has been demonstrated to contribute to subclinical atherosclerosis, it is considered to be a risk factor for the development of cardiovascular disease (Ortega et al., 2016). The prevalence of obesity is rapidly increasing. A recent study reported that the global prevalence of obesity increased from 3.2% in 1975 to 10.8% in 2014 in men, and from 6.4% to 14.9% in women. For obesity, China moved from 13^th^ place for men and 10^th^ for women in 1975, to 1^st^ rank for both men and women in 2014. Moreover, for severe obesity, China moved from 60^th^ place for men and 41^st^ for women in 1975, to 2^nd^ rank for both men and women in 2014. Nonetheless, more than one in four severely obese men and almost one in five severely obese women in the world still live in the USA. Rather, if present trends continue, by 2025, global obesity prevalence will reach 18% in men and surpass 21% in women; severe obesity will surpass 9% in women and 6% in men (Collaboration, 2016).

- 1. **Obesity and cardiovascular function**

A key step in the pathogenesis of atherosclerosis is impaired endothelial function, and it is positively correlated with obesity (Brook, 2006). A chief function of vascular endothelium is to regulate vascular smooth muscle tone. Using flow-mediated dilation (FMD), brachial artery diameter responses can be measured and used to evaluate endothelial function (Korkmaz and Onalan, 2008). Abnormalities in arterial function contribute to an increased risk of cardiovascular events in obese patients (Ortega et al., 2016). Previous studies have established that obesity is positively correlated with arterial stiffness, which is a key characteristic of atherosclerosis (Van Guilder et al., 2006; Acree et al., 2007). Pulse wave velocity (PWV) is known as an indicator of arterial stiffness. Aortic arterial stiffness is measured by carotid-femoral PWV (cfPWV), and systemic arterial stiffness is measured by brachial-ankle PWV (baPWV). These markers are considered to be simple and reliable indicators of conditions of central and systemic arterial function.

- 1. **Obesity and endothelial progenitor cells (EPCs)**

Recent studies suggest that an injured endothelial monolayer is regenerated by circulating bone marrow derived-EPCs, and thus, circulating EPC levels reflect the endothelial repair capacity (Koutroumpi et al., 2012). An altered status of circulating EPCs indicates endothelial dysfunction, and thus, the level of circulating EPCs could be used as a surrogate index of cumulative cardiovascular risk (Koutroumpi et al., 2012). An altered status of circulating EPCs indicates endothelial dysfunction, and thus, the level of circulating EPCs could be used as a surrogate index of cumulative cardiovascular risk (Koutroumpi et al., 2012). In fact, in 2008, Muller-Ehmsen et al. demonstrated for the first time that obesity is associated with a decreased number of circulating progenitor cells (Muller-Ehmsen et al., 2008). Further studies revealed evidence to support that there is a reduction in the number and function of EPCs in obese subjects (MacEneaney et al., 2009; Heida et al., 2010; Tobler et al., 2010).

- 1. **Effects of exercise on cardiovascular function**

Physical inactivity is one of the main risk factors for the development of several chronic degenerative diseases such as cardiovascular disease, diabetes, osteoporosis and some types of cancer. Active lifestyle is associated with less body fat, improved cardiovascular, bone and muscle function, reduced anxiety and depression (Weinsier et al., 1998).

Previous studies recognize that an improvement in endothelial function occurs in obese people by exercise (Sawyer et al., 2016) and diet intervention (Sasaki et al., 2002), and that combining both exercise and diet induces the most significant improvements (Woo et al., 2004).

Thereafter, research was done on the effect of physical activity on EPC mobilization, and it produced some interesting findings. For example, physical exercise had a positive effect on EPC mobilization by increasing both the number of EPCs and their functions (Van Craenenbroeck et al., 2010; Schlager et al., 2011; Cesari et al., 2012). However, the underlying mechanisms have not been elucidated.

1. **Objective**

To investigate the effects of an 8-week intervention program of a combination of exercise and diet on cardiovascular function including endothelial function, the number and functions of EPCs, autonomic function and arterial stiffness in obese adults, and the underlying mechanisms.

1. **Method**
   1. **Study design**

**Participants**

Obese adults from a traditional weight loss camp will be recruited to this study. The closed weight loss camp has uniformly managed accommodations, a controlled diet, and required physical training during the 8-week intervention period. The campers will be housed in the same building and could not move in and out freely during their stay. The measurements will be conducted before and after the subjects completed this 8-week weight loss program of exercise and diet.

Informed consent will be obtained from each individual prior to the measurements. This study will be conducted according to the Declaration of Helsinki. And the study will obtain approval from the Ethics Committee of Guangzhou Sport University.

**Inclusion criteria**

Obese adults (aged 18 years and over) have a body mass index (BMI) ≥ 30 kg/m^2^.

**Exclusion criteria**

Unstable angina pectoris, myocardial infarction within the last 12 months, decompensated heart failure, cardiomyopathy, severe valvular heart disease, considerable pulmonary disease, uncontrolled hypertension, kidney failure, orthopaedic and/or neurological limitations to exercise, surgery during the intervention period, and drug or alcohol abuse.

**Diet intervention**

Subjects will be provided with energy-restricted diets of 1300-2200 kcal/day based on weight. The energy percentages provided by protein, fat and carbohydrate are 20%, 20% and 60% respectively, and energy distributions at breakfast, lunch and dinner are 30%, 40% and 30% respectively. All meals will be prepared and supervised by registered professional dietitians during the diet intervention.

**Exercise training**

Subjects will perform a training program 6 days/week for 8 weeks. The program is primarily comprised of endurance exercise such as bicycling, walking, running, dancing and ball games for 5 hours/day. It is also supplemented with resistance exercise. The endurance exercises involve moderate-intensity exercise training and high-intensity exercise training. The intensity of moderate-intensity training is set at 70-85% of the subject’s maximum heart rate (HRmax), which is calculated with the formula of 208 – (0.7×age). Heart rate will be continuously monitored by Polar heart rate monitors and recorded by researchers. The high-intensity exercise training (~90% of HRmax) is alternated with low-intensity exercise (~60% of HRmax) during the training. Resistance exercise is conducted with an intensity of 12-15 RM. The exercise program is designed to result in an energy expenditure of 1500-2500 kcal/day. Qualified trainers will supervise the subjects during the training program.

**Primary and secondary outcomes**

Primary outcome is endothelial function which is assessed by FMD. Secondary outcomes include anthropometry, resting heart rate, blood pressure, body composition, aerobic fitness, maximal strength, EPC number and function, arterial stiffness (cfPWV, baPWV, central blood pressure, and augmentation index (AIx)), cardiac autonomic function (standard deviation of normal-to-normal intervals (SDNN), square root of the mean squared differences of successive normal-to-normal intervals (RMSSD), total power (TF), low-frequency power in normalized units (LFnu), high-frequency power in normalized units (HFnu), and low-frequency power/high-frequency power (LF/HF)), and multiple biochemical markers (lipid profiles, insulin, tumor necrosis factor-alpha, high-sensitivity C-reactive protein, superoxide dismutase, vascular endothelial growth factor, endothelial nitric oxide synthase, adiponectin and irisin).

- 1. **Sample size**

Sample size is calculated based on 1% change in FMD after weight loss with an expected standard deviation in FMD from our laboratory of 1.3%. The sample size required for the study, with an α of 0.05 and β of 0.20 at 5% level of significance (two-sided) and estimating a refusal rate of 20%, is 20 subjects.

- 1. **Measurement procedures**

**Anthropometry and body composition**

Height and weight will be measured to calculate BMI (kg/m^2^). Body composition will be determined using a body composition analyzer (Inbody 370, Biospace, Seoul, Korea).

**Resting heart rate and brachial blood pressure**

Resting heart rate (HR) and brachial systolic / diastolic blood pressure (SBP / DBP) will be measured in triplicate using a sphygmomanometer while the subjects are seated for at least 10 min, and the average of the three readings will be recorded.

**Aerobic fitness**

Aerobic fitness will be assessed using the Physical Working Capacity test on a cycle ergometer (Ergoselect 100, Ergoline, Bitz, Germany) at a heart rate of 150 beats per minute (PWC150) or 170 beats per minute (PWC170). Heart rate will be measured with a Polar heart rate monitor. Prior to the test, subjects will be instructed to pedal with a steady cadence of 60 revolutions per minute. The start power is 50 watts (W) and is followed by a gradual rise of 25 W for PWC150 or 50 W for PWC170 every 2 min until the targeted heart rate (150 bpm or 170 bpm) is achieved and maintained at the steady state. PWC150 or PWC170 is calculated as the power relative to body mass (W/kg) corresponding to the heart rate of 150 bpm or 170 bpm, respectively.

**Maximum strength test (1RM)**

The maximum strength is determined by the one repetition maximum (1RM) bench press test. The 1RM test will be administered after each subject performed two warm-up sets. The warm-up sets are a pyramid system of increasing weight and decreasing repetitions. After all warm-up sets are completed, the subject will then attempt the 1RM.

**Endothelial function**

Endothelial function will be assessed by brachial artery FMD using echography (UNEX-EF, UNEX Co. Ltd., Nagoya, Japan). The subjects will rest in the supine position for 30 min in a quiet air-conditioned room, and then the right brachial artery will be scanned in longitudinal sections 2-10 cm above the elbow. A pneumatic cuff will be placed around the forearm and will be inflated for 5 min to at least 50 mmHg above systolic pressure. The brachial artery will be scanned and its baseline diameter will be recorded before cuff inflation, and then continuously from the release point to 2 min after cuff deflation to obtain the maximum diameter during reactive hyperemia. FMD is calculated as the maximum percent increase in arterial diameter during continuous measurement of arterial diameter following cuff deflation. Subsequently, endothelium-independent dilation will be measured by administrating 500 µg of nitroglycerine (NTG) sublingually.

**Heart rate variability**

HRV is used to determine the autonomic activity. The subjects will rest in the supine position for at least 10 min before the data are collected using the SphygmoCor device (AtCor Medical, Sydney, Australia). As for the time domain analysis, we will determine the standard deviation (SD) of normal-to-normal (NN) intervals (SDNN) and square root of the mean squared differences of successive NN intervals (RMSSD). SDNN is a measure of overall HRV, so a lower SDNN level indicates reduced overall HRV. RMSSD is a measure of parasympathetic autonomic function, and a reduced RMSSD is a marker of parasympathetic loss. For frequency domain analysis, total power (TP) in the frequency (0-0.40 Hz) will be divided into low-frequency power (LF: 0.04-0.15 Hz, modulated mainly by sympathetic system) and high-frequency power (HF: 0.15-0.40 Hz, modulated by parasympathetic system). Normalized units (nu) will be calculated by dividing the power of LF or HF by (LF+HF). LF/HF ratio reflects the complex interplay between sympathetic and parasympathetic modulation (sympathovagal balance). Increased sympathovagal balance is considered to reflect predominant sympathetic activity.

**Pulse wave velocity**

cfPWV will be measured using the SphygmoCor device (AtCor Medical, Sydney, Australia). The distance from the suprasternal notch to the carotid artery site (d-carotid) and the distance from the suprasternal notch to the femoral artery site (d-femoral) will be measured in meters using a standard measuring tape. A pressure sensitive transducer (tonometer) will be used on the carotid and femoral arterial sites along with three electrocardiogram leads attached to the participants' chest in order to measure the transit time of the pulse wave from the left ventricle to the carotid arterial site (t1) and from the left ventricle to the femoral arterial site (t2), respectively. The cfPWV will be calculated as the difference in distance between d-femoral and d-carotid divided by the mean difference between t1 and t2 (12). baPWV and cfPWV will be also measured with an oscillometric device (boso ABI-system 100; BOSCH & SOHN, Germany).

**Pulse wave analysis**

Aortic pressure waveforms will be derived using a generalized validated transfer function (SphygmoCor, AtCor Medical, Sydney, Australia). The aortic pressure wave is composed of a forward wave, caused by stroke volume ejection, and a reflected wave that returns to the aorta from systemic sites. Augmentation pressure is the difference between the second and first systolic peaks. The augmentation index (AIx) will be defined as the augmentation pressure expressed as a percentage of the aortic pulse pressure.

**Blood markers**

A fasting venous blood sample will be collected into evacuated plastic tubes containing ethylenediaminetetraacetic acid (EDTA) before and after the 8-week program. Whole venous blood will be also collected in tubes without anticoagulant for serum preparation. Total cholesterol, triglycerides, high-density lipoprotein cholesterol (HDL-c), low-density lipoprotein cholesterol (LDL-c), fasting glucose, and fasting insulin will be measured. Insulin resistance will be evaluated using the Homeostasis Model of Assessment of Insulin Resistance (HOMA-IR) and will be calculated as [fasting insulin (µU/ml) × fasting glucose (mmol/L)] / 22.5. Circulating irisin levels will be measured using an irisin ELISA kit (Phoenix Pharmaceuticals, CA, USA), and the manufacturer’s instructions will be followed. Serum concentrations of vascular endothelial growth factor (VEGF), endothelial nitric oxide synthase (eNOS), adiponectin, tumor necrosis factor-alpha (TNF-α), high-sensitivity C-reactive protein (hsCRP), and superoxide dismutase (SOD) will be analyzed using ELISA Kits (Cusabio, Biotech. Co., LTD, Wuhan, China), and the manufacturer’s instructions will be followed for each kit.

**Flow cytometric quantification of EPCs**

Quantification of EPCs is defined as CD34^+^/KDR^+^ cells. EDTA-anticoagulated whole blood samples will be labeled with APC-labeled anti-human CD34 (eBioscience) and AlexaFlour488-labeled anti-human KDR (Biolegend) monoclonal antibodies. Fluorescent isotype-matched antibodies will be used as controls. EPCs will be quantitatively measured using a Cytomics FC500 flow cytometer (Beckman Coulter, USA), and acquisition will be stopped after 40,000 events. Data will be analyzed using CXP software 2.0.

**EPC culture**

For EPC culture, 10 ml of peripheral blood will be obtained from obese subjects, and total mononuclear cells will be isolated by density gradient centrifugation (400 g for 30 min) with Histopaque-1077 (Sigma, St. Louis, MO, USA). Then mononuclear cells will be cultured on fibronectin-coated 6-well plates in M199 medium supplemented with 20% FBS, 100 U/ml penicillin, 100 µg/ml streptomycin, and 10 ng/ml VEGF. After 4 days in culture, nonadherent cells will be removed. Adherent cells will be maintained until day 7 and then used for EPC functional assays.

**EPC migration assay**

For EPC migration assay, a total of 2×10^4^ isolated EPCs will be resuspended in 250 µl serum-free M199 medium and placed in the upper chamber of a modified Boyden chamber (Costar Transwell assay, 8 µm pore size; Corning, NY, USA). The chamber will be placed in a 24-well culture dish containing 500 µl M199 medium supplemented with 10 ng/ml VEGF. After a 24-hour incubation at 37˚C, the membrane will be washed briefly with phosphate-buffered saline (PBS) and fixed with 4% paraformaldehyde. The membrane will be then stained using 0.1% crystal violet solution and carefully removed. The transmigrated cells will be counted manually in three random microscopic fields (×200) by independent, blinded investigators.

**EPC adhesion assay**

For the EPC adhesion assay, EPCs will be isolated and resuspended in M199 medium with 5% FBS. An equivalent amount of cells will be placed on a fibronectin-coated 96-well plate and incubated at 37˚C in 5% CO_2_ for 6 hours. After two gentle washes with PBS, adherent cells will be counted by independent, blinded investigators.

- 1. **Data analysis**

Analyses will be performed using SPSS 16.0 (SPSS 16, SPSS Inc., Chicago, IL, USA). Paired-sample t-tests will be used to compare the effects of an 8-week intervention of exercise and diet on different variables. Pearson’s correlation will be calculated to determine associations. The descriptive analyses will calculate the mean and standard deviations for continuous and percentages for categorical variables. A P-value < 0.05 will be used to indicate statistical significance.

•

•

•

•

1. **References**

Acree, L.S., Comp, P.C., Whitsett, T.L., Montgomery, P.S., Nickel, K.J., Fjeldstad, A.S., et al. (2007). The influence of obesity on calf blood flow and vascular reactivity in older adults. *Dyn Med* 6**,** 4. doi: 10.1186/1476-5918-6-4.

Brook, R.D. (2006). Obesity, weight loss, and vascular function. *Endocrine* 29(1)**,** 21-25. doi: 10.1385/ENDO:29:1:121.

Cesari, F., Sofi, F., Gori, A.M., Corsani, I., Capalbo, A., Caporale, R., et al. (2012). Physical activity and circulating endothelial progenitor cells: an intervention study. *Eur J Clin Invest* 42(9)**,** 927-932. doi: 10.1111/j.1365-2362.2012.02670.x.

Collaboration, N.C.D.R.F. (2016). Trends in adult body-mass index in 200 countries from 1975 to 2014: a pooled analysis of 1698 population-based measurement studies with 19.2 million participants. *Lancet* 387(10026)**,** 1377-1396. doi: 10.1016/S0140-6736(16)30054-X.

Heida, N.M., Muller, J.P., Cheng, I.F., Leifheit-Nestler, M., Faustin, V., Riggert, J., et al. (2010). Effects of obesity and weight loss on the functional properties of early outgrowth endothelial progenitor cells. *J Am Coll Cardiol* 55(4)**,** 357-367. doi: 10.1016/j.jacc.2009.09.031.

Korkmaz, H., and Onalan, O. (2008). Evaluation of endothelial dysfunction: flow-mediated dilation. *Endothelium* 15(4)**,** 157-163. doi: 10.1080/10623320802228872.

Koutroumpi, M., Dimopoulos, S., Psarra, K., Kyprianou, T., and Nanas, S. (2012). Circulating endothelial and progenitor cells: Evidence from acute and long-term exercise effects. *World J Cardiol* 4(12)**,** 312-326. doi: 10.4330/wjc.v4.i12.312.

MacEneaney, O.J., Kushner, E.J., Van Guilder, G.P., Greiner, J.J., Stauffer, B.L., and DeSouza, C.A. (2009). Endothelial progenitor cell number and colony-forming capacity in overweight and obese adults. *Int J Obes (Lond)* 33(2)**,** 219-225. doi: 10.1038/ijo.2008.262.

Muller-Ehmsen, J., Braun, D., Schneider, T., Pfister, R., Worm, N., Wielckens, K., et al. (2008). Decreased number of circulating progenitor cells in obesity: beneficial effects of weight reduction. *Eur Heart J* 29(12)**,** 1560-1568. doi: 10.1093/eurheartj/ehn213.

Ortega, F.B., Lavie, C.J., and Blair, S.N. (2016). Obesity and Cardiovascular Disease. *Circ Res* 118(11)**,** 1752-1770. doi: 10.1161/CIRCRESAHA.115.306883.

Sasaki, S., Higashi, Y., Nakagawa, K., Kimura, M., Noma, K., Sasaki, S., et al. (2002). A low-calorie diet improves endothelium-dependent vasodilation in obese patients with essential hypertension. *Am J Hypertens* 15(4 Pt 1)**,** 302-309.

Sawyer, B.J., Tucker, W.J., Bhammar, D.M., Ryder, J.R., Sweazea, K.L., and Gaesser, G.A. (2016). Effects of high-intensity interval training and moderate-intensity continuous training on endothelial function and cardiometabolic risk markers in obese adults. *J Appl Physiol (1985)* 121(1)**,** 279-288. doi: 10.1152/japplphysiol.00024.2016.

Schlager, O., Giurgea, A., Schuhfried, O., Seidinger, D., Hammer, A., Groger, M., et al. (2011). Exercise training increases endothelial progenitor cells and decreases asymmetric dimethylarginine in peripheral arterial disease: a randomized controlled trial. *Atherosclerosis* 217(1)**,** 240-248. doi: 10.1016/j.atherosclerosis.2011.03.018.

Tobler, K., Freudenthaler, A., Baumgartner-Parzer, S.M., Wolzt, M., Ludvik, B., Nansalmaa, E., et al. (2010). Reduction of both number and proliferative activity of human endothelial progenitor cells in obesity. *Int J Obes (Lond)* 34(4)**,** 687-700. doi: 10.1038/ijo.2009.280.

Van Craenenbroeck, E.M., Hoymans, V.Y., Beckers, P.J., Possemiers, N.M., Wuyts, K., Paelinck, B.P., et al. (2010). Exercise training improves function of circulating angiogenic cells in patients with chronic heart failure. *Basic Res Cardiol* 105(5)**,** 665-676. doi: 10.1007/s00395-010-0105-4.

Van Guilder, G.P., Hoetzer, G.L., Dengel, D.R., Stauffer, B.L., and DeSouza, C.A. (2006). Impaired endothelium-dependent vasodilation in normotensive and normoglycemic obese adult humans. *J Cardiovasc Pharmacol* 47(2)**,** 310-313. doi: 10.1097/01.fjc.0000205097.29946.d3.

Weinsier, R.L., Hunter, G.R., Heini, A.F., Goran, M.I., and Sell, S.M. (1998). The etiology of obesity: relative contribution of metabolic factors, diet, and physical activity. *Am J Med* 105(2)**,** 145-150.

Woo, K.S., Chook, P., Yu, C.W., Sung, R.Y., Qiao, M., Leung, S.S., et al. (2004). Effects of diet and exercise on obesity-related vascular dysfunction in children. *Circulation* 109(16)**,** 1981-1986. doi: 10.1161/01.CIR.0000126599.47470.BE.
