# Supplementary material for: Exercise training with dietary restriction enhances circulating irisin level associated with increasing endothelial progenitor cell number in obese adults: an intervention study
Source: PeerJ. 2017 Aug 14;5:e3669. doi: 10.7717/peerj.3669 (PMC5560232; doi:10.7717/peerj.3669)
Supplement: Supplemental Information 2 [file peerj-05-3669-s002.doc]

**标题：运动训练与饮食控制对肥胖人群心血管功能的影响**

**短标题：运动，饮食和心血管功能**

**方案编号：Trial2016.1 Version 1.0**

**项目单位：广州体育学院**

**广州市广州大道中1268号**

**项目代理人：王丹**

**广州市广州大道中1268号广州体育学院科研楼1楼**

**+86 (0)20 3802 5087**

**17880383@qq.com**

**项目医学专家：黄俊豪**

**广州市广州大道中1268号广州体育学院科研楼1楼**

**020-38025087**

**junhaohuang2006@hotmail.com**

**项目负责人：胡敏**

**广州市广州大道中1268号广州体育学院**

**020-38024222**

**whoomin@hotmail.com**

**试验地点：惠州市阳光光亚减肥训练营**

**1.研究背景**

**1.1．肥胖发生趋势**

肥胖（obesity）是引起血脂紊乱、II型糖尿病、高血压和动脉粥样硬化等心血管疾病（CVD）的重要危险因素 (Ortega et al., 2016)。今年4月发表在柳叶刀杂志上的一份涵盖186个国家的研究发现，40年来，全球男性肥胖率从3.2%升至10.8%，女性肥胖率则从6.4%升至14.9%。调查还指出，中国的肥胖人口位居世界首位，分别占全球的16.3%和12.4%；而在严重肥胖的人口中，中国从1975年男性和女性分列第60和第41位升至第二，仅次于美国。研究称，如果现下这种趋势持续下去，那么到2025年，全球男性肥胖率将达到18%，女性肥胖率将超过21%，而同期男性和女性的严重肥胖率将分别超过6%和9% (Collaboration, 2016)。

**1.2．肥胖与心血管功能的关系**

血管功能障碍是动脉硬化的基础，许多心血管疾病及危险因素可导致内皮功能损伤，而内皮功能损伤也常常伴发和加重心血管疾病，后者又与肥胖关系密切(Brook, 2006)。然而，除了已知的心血管危险因素，系统的炎症增加、氧化应激而且体重增加改变的血流动力学可能直接导致血管内皮损伤与功能障碍，可能是肥胖者动脉粥样硬化的发病机理。通过超声测量肱动脉血流介导的血管舒张(flow-mediated dilation, FMD)评估血管内皮功能(Korkmaz and Onalan, 2008)以及通过检测脉搏波传导速度（pulse wave velocity, PWV）判断动脉硬化的方法(Van Guilder et al., 2006; Acree et al., 2007)，越来越多地用于心血管功能特别是内皮功能的风险评估，逐渐成为CVD初级预防和早期治疗的新策略。因此，临床上检测FMD和PWV，可以更准确、有效地评估运动对超重或肥胖人群血管功能包括内皮功能的影响。

**1.3．肥胖与EPC的关系**

内皮祖细胞（EPC）在维持血管内皮层的完整性发挥重要的作用(Koutroumpi et al., 2012)。它具有缺血区定向归巢并分化为成熟内皮细胞、迟发高增殖潜能等特性，不仅参与胚胎发育时的血管发生，在出生后也起着促进受损内皮修复、微血管新生等重要作用。

大量研究表明，肥胖与EPC的数量呈负相关(Muller-Ehmsen et al., 2008)。例如，MacEneaney等在对正常体重、超重和肥胖成人的比较中发现，肥胖人群中EPC的数量低于前两者，而EPC集落形成能力在超重及肥胖者要弱于体重正常人群，提示EPC数量和功能的受损可能是肥胖导致相关心血管疾病风险上升的机制之一(MacEneaney et al., 2009; Heida et al., 2010; Tobler et al., 2010)。

**1.4.运动对心血管功能的影响**

大量研究证实，运动有助于改善血管内皮功能(Weinsier et al., 1998)，此外，饮食也能改善肥胖人群血管内皮功能(Sasaki et al., 2002)，两者结合的效果最明显(Woo et al., 2004)。近年来，越来越多的研究开始关注运动对EPC的影响，这可能是运动改善内皮功能的作用机制之一(Van Craenenbroeck et al., 2010; Schlager et al., 2011; Cesari et al., 2012)。

**2.研究目的**

8周运动和饮食干预对肥胖受试者心血管功能包括血管内皮功能、EPC数量和功能、自主神经系统和动脉硬化的影响，以及其中的内在机理。

**3.研究方法**

**3.1.研究设计**

**受试者**

本研究拟选取参加运动减肥训练营的成年肥胖者（年龄大于18岁，BMI > 30kg/m^2^）为研究对象。剔除标准包括不稳定心绞痛，1年内发生过心肌梗塞，失代偿心衰竭，心肌病，严重的心瓣膜疾病，严重肺部疾病，不受控的高血压，肾衰竭，整形手术或者神经疾病导致运动受限，运动干预期进行手术，药物或酒精滥用等。所有受试者均要求预先了解整个实验流程及目的，整个实验过程中可随时自由退出实验，填写知情同意书。

运动干预期间所有受试者的饮食均为经过营养师调配的低盐低脂饮食, 平均每日摄入的食物量热量大概在1300-2200kcal之间。早、中及晚餐提供的热量比例约为30%，40%，30%，其中糖、脂肪及蛋白质的供能量约占总热量的60%，20%及20%，严格统一作息制度。

所有受试者每周训练6天，休息1天，共训练8周；每天训练5小时，上午下午各2.5小时，训练内容以中高强度的有氧运动以及高强度的间歇训练为主，每天的训练课程平均热量消耗约在1500-2500kcal之间。中高强度的有氧训练以70%～85%最大心率进行训练；高强度间歇训练采用高强度期间（如以90%的最大心率进行运动）和低强度期间（如以60%最大心率进行运动）来回交替；此外，受试者也会进行一些抗阻力量训练，负荷一般为12～15RM。训练由专业教练带动受试者共同完成课程训练任务。

**首要指标和次要指标**

首要指标是血管内皮功能（FMD值）。次要指标包括身高、体重、体成分、安静心率、收缩压、舒张压、有氧能力、最大力量、中心动脉压、中心动脉压差、主动脉溢压、心脏负荷指数和心率变异性（HRV）；血液生化指标：总甘油三酯 （ total triglyceride, TG）、总胆固醇（total cholesterol, TC）、高密度脂蛋白（high-density lipoprotein cholesterol, HDL-C）、低密度脂蛋白（low-density lipoprotein cholesterol, LDL-C）、空腹血糖(FBG)、空腹血胰岛素（FINS）；炎症相关指标：Ｃ-反应蛋白（CRP）、肿瘤坏死因子-α（TNF-α）；氧化应激相关指标：超氧化物歧化酶（SOD）；血管功能指标：FMD、cfPWV、baPWV、外周血的eNOS、VEGF和EPC含量；EPC数量和功能。

**3.2.样本量计算**

根据我们实验室的预实验结果，设定运动干预后首要指标FMD的观测比较阈值为1%，标准差为1.3%；设定α为0.05,效能为80%，在G-power中计算出最小样本量，考虑到样本的依从率和流失情况（20%），最后确定肥胖训练人数为每组20人。

**3.3.测量方法**

**身体基本情况测试**

对所有研究对象进行身高、体重、腰臀比、BMI、安静心率、血压等指标的测试；并采用体成分测试仪对所有研究对象的体成分进行测试。

**心血管功能检测**

使用心血管功能检测仪（型号：EM3/MM3；AtCor；澳大利亚）检测中心动脉压（CAP）、中心动脉压差（CPP）、主动脉溢压（AP）、心脏负荷指数（AIx）和心率变异性（HRV）。该仪器测量桡动脉或颈动脉的脉搏信号，同时测量肱动脉血压，使用经过验证的通用的转换公式可估算出中心血压。与直接测量的动脉内血压进行的比较研究证实，SphygmoCor计算出的中心动脉压基本准确，可以作为评价心血管风险的评价指标。

**血管内皮功能检测**

采用UNEX EF血管内皮功能检测仪(UNEX-EF, UNEX Co. Ltd., Nagoya, Japan)，对受试者肱动脉血流介导舒张功能(FMD)进行测定。UNEXEF是专门用于血管内皮功能检测的超声检测仪，能自动敏锐的捕捉出血管内径的实时变化。根据血管和短轴传感器的位置关系，可以掌握探头的调整方向，并且通过微型电动机可以自动微调整探头方向，仪器通过其内置的软件自动计算出肱动脉内径的变化率。测试时间统一安排在上午8-10点进行，受试者处于仰卧位，选取其右侧肘关节上方2-10cm处的肱动脉。

**脉搏波传导速度（PWV）检测**

PWV是由心脏搏动射血而引起脉搏波动的传导速度，通过此项指标来判断动脉弹性的状况，管壁越硬脉搏波传导速度越快。PWV是评价动脉硬度的经典指标。cfPWV是颈股动脉间脉搏波传导速度，是衡量中心大动脉血管弹性和动脉硬化情况的指标，是目前检测大动脉硬度的金标准。cfPWV增大提示主动脉硬度增高，cfPWV越大，主动脉的损害越严重。我们采用心血管功能检测仪（型号：EM3/MM3；AtCor；澳大利亚），可以测量颈股动脉脉搏波传导速度（cfPWV）、踝臂脉搏波传导速度（baPWV）、踝臂指数（ABI）、左右手臂血压差，并自动计算各重要指标。

**生化指标测试**

分别在运动干预前、后收集空腹状态下的肘静脉血，分离血清置-80^o^C保存待测。脂代谢指标采用酶比色法测定：包括TG、TC、HDL-C、LDL-C；氧化应激指标包括SOD，采用酶联免疫吸附法测定；炎症指标包括CRP、TNF-α,均采用酶联免疫吸附法测定；eNOS、VEGF、Irisin采用酶联免疫吸附法测定；血糖（FBG）采用氧化酶法测定，血胰岛素（FINS）水平采用酶联免疫吸附法测定，并计算胰岛素抵抗指数：HOMA-IR=FBG x FINS/22.5。

**外周血EPC数量的检测**

取血5ml，放置于含有EDTA的管中，血细胞用荧光素标记的抗人单克隆抗体，包括AlexaFlour488标记的CD309（VEGFR-2/KDR）(Biolegend公司)和APC标记的CD34（eBioscience公司）。按照抗体说明书，血液样品（200ul）室温下在抗体中孵育30min。相应的同种型匹配的抗体作为阴性对照。抗体温育后，将各样品用2.5ml的1X裂解液10min(BD Biosciences公司)，然后用FACS缓冲液洗涤两次。每个样品在Cytomics FC500流式细胞仪(Beckman Coulter公司)检测，上样量为4X10^5^events，后用CXP software 2.0进行分析。

**外周血EPC的分离、培养以及EPC迁移和粘附能力检测**

采集肥胖成年人新鲜的抗凝外周血10 ml,分离单个核细胞(MNCs)。将MNCs 接种在HFN (5µg/cm^2^)包被的6孔培养板上,置于37℃、5%的CO_2_、饱和湿度的CO_2_培养箱中培养。细胞培养4天后,去除未附着细胞,继续培养粘附细胞至第7天,倒置显微镜观察其生长情况。

**EPC迁移和粘附能力检测**

在获得理想的EPC后，按照文献的方法检测EPC迁移和粘附的能力，比较肥胖对EPC功能活性的改变，以及培养基孵育一定浓度的Irisin对EPC功能活性的影响。

**3.4.数据统计学处理**

**采用SPSS 22.0软件对数据进行统计分析，数据用均数±标准差表示。运动前、后比较采用配对t检验。两变量前、后变化值的相关性采用等级相关分析（pearson），检测水平为P < 0.05。**

**4.参考文献**

Acree, L.S., Comp, P.C., Whitsett, T.L., Montgomery, P.S., Nickel, K.J., Fjeldstad, A.S., et al. (2007). The influence of obesity on calf blood flow and vascular reactivity in older adults. *Dyn Med* 6**,** 4. doi: 10.1186/1476-5918-6-4.

Brook, R.D. (2006). Obesity, weight loss, and vascular function. *Endocrine* 29(1)**,** 21-25. doi: 10.1385/ENDO:29:1:121.

Cesari, F., Sofi, F., Gori, A.M., Corsani, I., Capalbo, A., Caporale, R., et al. (2012). Physical activity and circulating endothelial progenitor cells: an intervention study. *Eur J Clin Invest* 42(9)**,** 927-932. doi: 10.1111/j.1365-2362.2012.02670.x.

Collaboration, N.C.D.R.F. (2016). Trends in adult body-mass index in 200 countries from 1975 to 2014: a pooled analysis of 1698 population-based measurement studies with 19.2 million participants. *Lancet* 387(10026)**,** 1377-1396. doi: 10.1016/S0140-6736(16)30054-X.

Heida, N.M., Muller, J.P., Cheng, I.F., Leifheit-Nestler, M., Faustin, V., Riggert, J., et al. (2010). Effects of obesity and weight loss on the functional properties of early outgrowth endothelial progenitor cells. *J Am Coll Cardiol* 55(4)**,** 357-367. doi: 10.1016/j.jacc.2009.09.031.

Korkmaz, H., and Onalan, O. (2008). Evaluation of endothelial dysfunction: flow-mediated dilation. *Endothelium* 15(4)**,** 157-163. doi: 10.1080/10623320802228872.

Koutroumpi, M., Dimopoulos, S., Psarra, K., Kyprianou, T., and Nanas, S. (2012). Circulating endothelial and progenitor cells: Evidence from acute and long-term exercise effects. *World J Cardiol* 4(12)**,** 312-326. doi: 10.4330/wjc.v4.i12.312.

MacEneaney, O.J., Kushner, E.J., Van Guilder, G.P., Greiner, J.J., Stauffer, B.L., and DeSouza, C.A. (2009). Endothelial progenitor cell number and colony-forming capacity in overweight and obese adults. *Int J Obes (Lond)* 33(2)**,** 219-225. doi: 10.1038/ijo.2008.262.

Muller-Ehmsen, J., Braun, D., Schneider, T., Pfister, R., Worm, N., Wielckens, K., et al. (2008). Decreased number of circulating progenitor cells in obesity: beneficial effects of weight reduction. *Eur Heart J* 29(12)**,** 1560-1568. doi: 10.1093/eurheartj/ehn213.

Ortega, F.B., Lavie, C.J., and Blair, S.N. (2016). Obesity and Cardiovascular Disease. *Circ Res* 118(11)**,** 1752-1770. doi: 10.1161/CIRCRESAHA.115.306883.

Sasaki, S., Higashi, Y., Nakagawa, K., Kimura, M., Noma, K., Sasaki, S., et al. (2002). A low-calorie diet improves endothelium-dependent vasodilation in obese patients with essential hypertension. *Am J Hypertens* 15(4 Pt 1)**,** 302-309.

Sawyer, B.J., Tucker, W.J., Bhammar, D.M., Ryder, J.R., Sweazea, K.L., and Gaesser, G.A. (2016). Effects of high-intensity interval training and moderate-intensity continuous training on endothelial function and cardiometabolic risk markers in obese adults. *J Appl Physiol (1985)* 121(1)**,** 279-288. doi: 10.1152/japplphysiol.00024.2016.

Schlager, O., Giurgea, A., Schuhfried, O., Seidinger, D., Hammer, A., Groger, M., et al. (2011). Exercise training increases endothelial progenitor cells and decreases asymmetric dimethylarginine in peripheral arterial disease: a randomized controlled trial. *Atherosclerosis* 217(1)**,** 240-248. doi: 10.1016/j.atherosclerosis.2011.03.018.

Tobler, K., Freudenthaler, A., Baumgartner-Parzer, S.M., Wolzt, M., Ludvik, B., Nansalmaa, E., et al. (2010). Reduction of both number and proliferative activity of human endothelial progenitor cells in obesity. *Int J Obes (Lond)* 34(4)**,** 687-700. doi: 10.1038/ijo.2009.280.

Van Craenenbroeck, E.M., Hoymans, V.Y., Beckers, P.J., Possemiers, N.M., Wuyts, K., Paelinck, B.P., et al. (2010). Exercise training improves function of circulating angiogenic cells in patients with chronic heart failure. *Basic Res Cardiol* 105(5)**,** 665-676. doi: 10.1007/s00395-010-0105-4.

Van Guilder, G.P., Hoetzer, G.L., Dengel, D.R., Stauffer, B.L., and DeSouza, C.A. (2006). Impaired endothelium-dependent vasodilation in normotensive and normoglycemic obese adult humans. *J Cardiovasc Pharmacol* 47(2)**,** 310-313. doi: 10.1097/01.fjc.0000205097.29946.d3.

Weinsier, R.L., Hunter, G.R., Heini, A.F., Goran, M.I., and Sell, S.M. (1998). The etiology of obesity: relative contribution of metabolic factors, diet, and physical activity. *Am J Med* 105(2)**,** 145-150.

Woo, K.S., Chook, P., Yu, C.W., Sung, R.Y., Qiao, M., Leung, S.S., et al. (2004). Effects of diet and exercise on obesity-related vascular dysfunction in children. *Circulation* 109(16)**,** 1981-1986. doi: 10.1161/01.CIR.0000126599.47470.BE.
